# Clinical and Psychological Factors to Consider in Achieving Treatment-Free Remission in Patients With Chronic Myeloid Leukemia

**DOI:** 10.3389/fonc.2021.631570

**Published:** 2021-03-10

**Authors:** Massimo Breccia, Elisabetta Abruzzese, Mario Annunziata, Luigia Luciano, Simona Sica

**Affiliations:** ^1^ Hematology, Department of Precision and Translational Medicine, Policlinico Umberto 1, Sapienza University, Rome, Italy; ^2^ Division of Hematology, Ospedale S. Eugenio, Tor Vergata University, Roma, Italy; ^3^ Hematology Division, Azienda Ospedaliera di Rilievo Nazionale Cardarelli, Naples, Italy; ^4^ Hematology - Department of Clinical Medicine and Surgery, Federico II University, Napoli, Italy; ^5^ Dipartimento di Diagnostica per Immagini, Radioterapia Oncologica ed Ematologia, Fondazione Policlinico Universitario A. Gemelli, Istituto di Ricovero e Cura a Carattere Scientifico, Roma, Italy; ^6^ Sezione di Ematologia, Dipartimento di Scienze Radiologiche ed Ematologiche, Università Cattolica del Sacro Cuore, Roma, Italy

**Keywords:** chronic myeloid leukemia, treatment-free remission, management, tyrosine kinase inhibitor, major molecular response

## Abstract

Treatment of chronic myeloid leukemia (CML) has evolved dramatically in recent years. In this regard, the introduction of second-generation tyrosine kinase inhibitors (TKI) has revolutionized therapeutic goals, and it is now desirable to obtain treatment-free remission (TFR), i.e. when a patient who has stopped TKI therapy maintains a major molecular response and does not need to restart treatment. This report summarizes the main findings from a group of expert hematologists in Italy who met to discuss treatment and management of patients with CML with focus on broad-ranging aspects of TFR. A survey was used to obtain information about the clinicians’ experience with TFR and to better understand the clinical and psychological issues that patients and physicians face when considering TFR. The overall goal was to explore the possibility of discontinuing treatment from multiple points of view, considering both clinical aspects of TFR as well as psychological management of patients. Practical information is provided on aspects associated with initiating TFR, clinical data supporting it, the role of monitoring, and management of discontinuation-related adverse events. This publication outlines many of the shortcomings and highlights proposed solutions for routine clinical practice, and provides an overview of the literature relative to TFR.

## Introduction

Treatment of chronic myeloid leukemia (CML) has evolved dramatically in recent years. In this regard, the introduction of second-generation tyrosine kinase inhibitors (TKI) has now revolutionized therapeutic goals. While in the past the goal for patients with CML was limited to hematological control of disease in the long term, for many patients it is now desirable to obtain treatment-free remission (TFR). TFR occurs when a patient who has stopped TKI therapy maintains a major molecular response (MMR) and does not need to restart treatment. Patients in the chronic phase who have maintained a deep MR (DMR) that is stable for at least 2 years are considered to be good candidates for discontinuation of TKI therapy. After discontinuation of a TKI, overt recurrence is considered when loss of MMR is confirmed (1).

From the patient’s point of view, TFR is desirable for several reasons: i) avoid the long-term effects of TKIs; ii) obtain a feeling of cure; iii) avoid intolerance not only to adverse effects, but also to maintain the therapeutic regimen; iv) special conditions such as pregnancy.

In this regard, controlled clinical studies have shown that it is possible to discontinue imatinib, as shown in the STIM (2) and ISAV trials (3), as well as for second-generation tyrosine kinase inhibitors in the EURO-SKI study (4) and nilotinib in selected cases (5). Accordingly, it has been requested that regulatory authorities modify the Summary of Product Characteristics for nilotinib to include the possibility of safely discontinuing the drug in selected patients who meet specific criteria. To date, however, at least in most centers in Italy, TFR is attempted in a limited number of patients who present specific indications, such as drug toxicity or pregnancy, in addition to those expressing the desire to discontinue the drug. Moreover, the objective clinical and psychological factors that can influence the propensity of the physician to propose and start TFR are very complex, and for this reason proposal of TFR is still limited in routine clinical practice. In any case, patients should be informed that periods of TFR can last from several months to many years and that all patients who need to restart therapy are able to regain and maintain the previous MR.

With these considerations in mind, a group of 25 hematologists from the Lazio, Campania and Abruzzo regions in Italy met to discuss treatment and management of patients with CML with focus on broad-ranging aspects of TFR. During the meeting a survey was used to obtain information about the experience of the clinicians with TFR as well as to better understand the clinical and psychological issues that patients and physicians face when considering the possibility to attempt TFR. Two plenary meetings were used to review the results of the survey, define points for discussion, overview the relevant literature and summarize recommendations for practice based on shared experience. The overarching aim of the project was to explore the possibility of discontinuing treatment from multiple points of view, considering not only the clinical aspects of TFR, but also psychological management of patients. Toward this end, a set of shortcomings and proposed solutions for routine clinical practice are offered as the main output of the project, along with an overview of the literature relative to TFR.

## Issues Associated With Initiating Treatment-Free Remission

**Figure 1:**
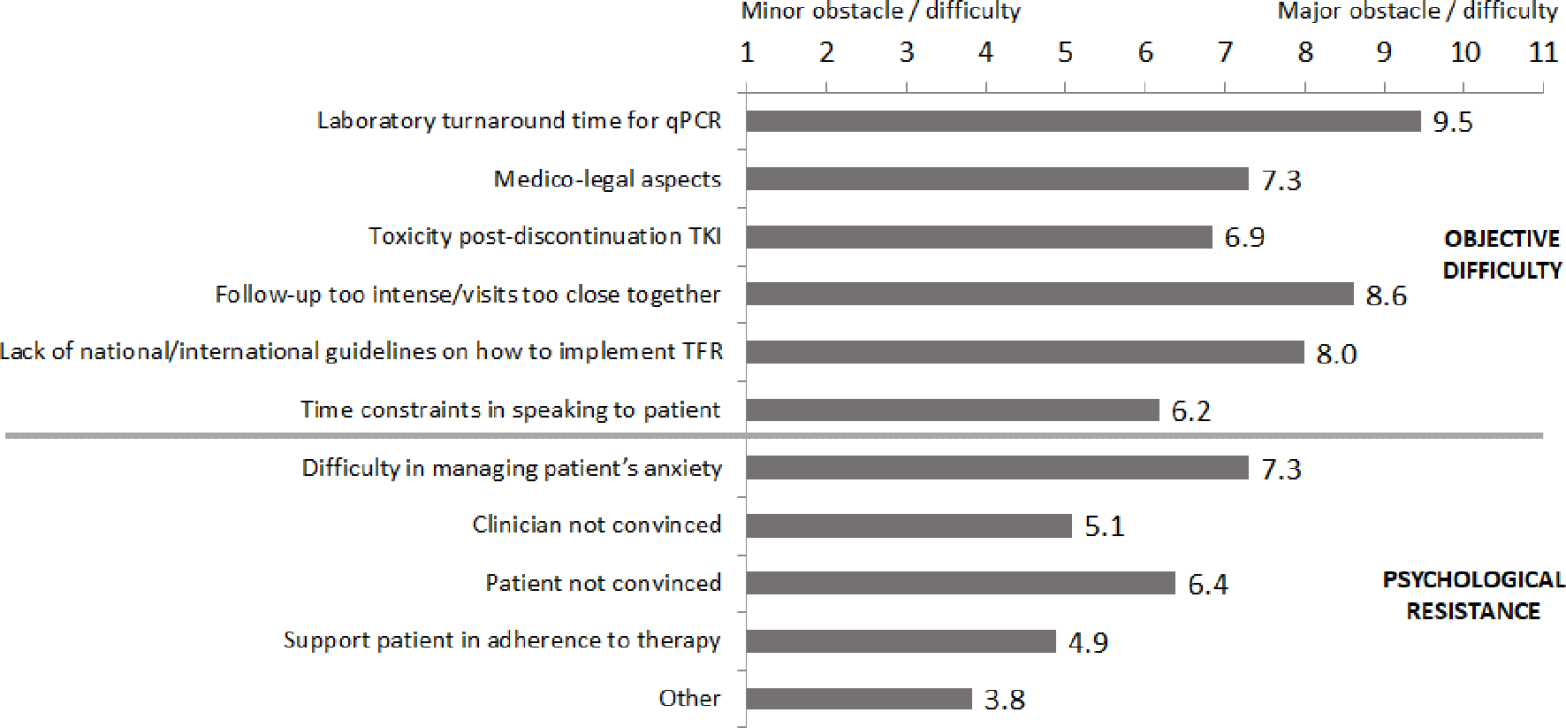
Obstacles and difficulties cited in proposing treatment-free remission to patients. qPCR, quantitative polymerase chain reaction; TKI, tyrosine kinase inhibitor.

An ad-hoc survey was used to query the participating physicians about the clinical and psychological issues perceived when considering initiating TFR (**Figure 1**). Overall, 63% of participants referred difficulty in proposing TFR to patients for both objective clinical as well as for potential psychological issues. Among clinical issues, the most widely cited were laboratory turnaround times needed for qPCR, the need for an intense molecular follow-up schedule (at least every month for the first 6 months after discontinuation) and the absence of published national and international guidelines on TFR. Among psychological issues, the most commonly mentioned were difficulty in managing the patient’s anxiety about the discontinuation proposal and the lack of conviction about TFR by both the patient and physician.

### Proposals for Overcoming Issues Associated With Initiating Treatment-Free Remission

Having identified some of the main obstacles associated with proposing TFR to patients, much time was dedicated during the plenary sessions on deciding how these obstacles can be overcome. The main suggestions provided for clinical issues are listed in **Table 1**. Regarding the possible issue of long turnaround times for tests, it was suggested, for example, that the molecular laboratory should be contacted directly to indicate the urgent nature of the test and that priority should be given since the results are needed for a patient in TFR.

**Table 1:** Clinical and psychological issues and proposed solutions.

	Comments	Proposed solutions
Clinical issues		
Problems related to laboratory analysis	This is an important clinical issue considering monitoring of molecular response: in some centers, reporting requires lengthy time intervals and is of potentially poor quality. This aspect did not emerge only at secondary centers that often rely on other laboratories, but also at large centers which are often overwhelmed by the large number of tests.	Personally contact the laboratory to indicate the urgency of the report; indicate that the test is urgent since it is needed for a TFR, possibly giving priority over tests on stable patients.
Medical-legal aspects	Fear of legal action by patients in case of relapse after suspension.	Emphasize to the patient that the possibility of TFR may be foreseen in the Summary of Product Characteristics. (To date, nilotinib is the only TKI that has a specific indication for TFR).	
Lack of guidelines	An important aspect for less experienced physicians.	Italian guidelines are being developed, pending those of the National Comprehensive Cancer Network (NCCN) and recommendations of Gruppo Italiano Malattie EMatologiche dell’Adulto (GIMEMA) (6)	
**Psychological issues**			
How to propose TFR	Clinicians may dissociate themselves from the choice of TFR: verbally and non-verbally the patient may feel abandoned. The clinician may not appear to be convinced of TFR, e.g. shows no confidence in answering the patient’s questions feels under pressure when faced with the patient’s questions or avoids answering them, is evasive, does not provide reassurance in response to the patient’s questions	TFR must be seen to be within the overall course of treatment proposed to the patient. This is the preferred solution to achieve greater awareness of therapy, as well as further stimulus to good adherence. This is especially useful in young patients with a higher socio-cultural profile	
Refusal of TFR by the patient	A clinician may complain about the inability to implement TFR because the patient refuses or is too anxious. This is one of the most frequent reasons and which in many cases is related to the clinician’s poor ability to reassure the patient. The physician agrees with the patient regarding fear of recovery of disease in case of discontinuation.	TFR can be proposed as a trial period for discontinuation to determine if it is possible to maintain the major molecular response in the absence of treatment, with the prerequisite that more intense follow-up is needed. In fact, it is not a question of abandoning the patient, but monitoring them more frequently.	
Management of “failure” to achieve the criteria for TFR	The clinician may have difficulty in communicating to the patient that they do not meet the criteria for TFR. This frustration can, in fact, become a deterrent in proposing TFR, to the extent that in some cases and in anxious or very demanding patients, the clinician fails to propose TFR.	Immediately clarify that only some patients meet criteria for TFR, and of these only about 50% will maintain the response.	
Need for close monitoring	The need for more intense follow-up can a critical aspect for the patient, as it may potentially conflict with the patient’s desire to feel less ill and discontinue therapy.	Immediately stress the need for more intense follow-up, and present it as a reassuring tool.With closer monitoring, it is possible to intervene promptly to prevent the loss of molecular response.	
Management of failed TFR	In terms of psychological management, the failure of TFR is a very delicate moment for the both patient and physician	In addition to proposing TFR as a trial period for discontinuation, it is important to clarify that the possibility of loss of major molecular response during TFR occurs in a significant proportion of patients and that this does not indicate return of disease.	

**Table 1** lists the psychological issues identified and their possible solutions. In general, among the solutions proposed, physicians should show greater confidence in TFR, and offer it in a more positive light. At the same time, patients should view TFR as part of the overall treatment strategy and as a period during which strict follow-up will be needed. Moreover, TFR should not be seen as something that all patients can achieve, and, importantly, that loss of MMR does not indicate that the disease has returned.

## Clinical Data in Support of Treatment-Free Remission

The main clinical data in support of TFR is briefly summarized below, and the reader is also referred to the proposal from the GIMEMA CML working party (7) and the European LeukemiaNet 2020 recommendations for treating CML (8). The most recent data are from the EURO-SKI study in which the MR-free survival (MRFS) curves indicate that the majority of patients experience recurrence within the first 6 months of discontinuation of therapy (4, 9). At 36 months, 47% of patients maintain MR. Similar results on MRFS were reported in the TWISTER study (45%) (10).

For nilotinib, two studies have demonstrated long-term maintenance of TFR: ENESTop investigated TFR after interruption of second-line nilotinib, while ENESTfreedom examined discontinuation of first-line nilotinib. In particular, 46% of patients in ENESTop and 44.2% in ENESTfreedom were still in TFR at 192 weeks following discontinuation (>3 years and 8 months) (6, 11–13). In addition, interim analysis of the DASFREE study, even if biased since it analyzed patients treated with dasatinib either in first and in second or subsequent lines of therapy, indicated that 63% of patients maintain TFR at 12 months (14).

### Restarting Treatment Allows Regaining Major Molecular Response

Even if about half of patients who discontinued TKIs resume treatment sooner or later, almost all achieve and maintain a MMR. This is another aspect that should reassure clinicians in proposing TFR. For example, the DADI and STIM2 studies indicate that, in case of loss of MMR, restarting TKI therapy allows the patient to regain and maintain a major response over time in just a few months (15, 16).

### Second Attempt at Treatment-Free Remission

The failure of a first attempt at TFR does not preclude the success of a second attempt. The observational RE-STIM study, carried out in patients who had regained a MR4.5 response after a previous attempt at TFR, showed that a second attempt at TFR is possible and safe (17). Molecular response MR4.0 is defined as BCR-ABL^IS^ ratio 0.01% (ABL1 transcripts ≥10,000), MR4.5 as BCR-ABL^IS^ ratio 0.0032% (ABL1 transcripts ≥32,000) and MR5.0 as BCR-ABL^IS^ 0.001% (ABL1 transcripts ≥100,000) (18). DMR is usually defined as ≥MR4.0. The probability of maintaining TFR after a second attempt was 42% at 24 months and 35% at 36 months. The study also showed that MR at 3 months after re-administration of the drug was a fundamental parameter to predict the success of second attempt at TFR. Most recently, the post-STIM trial has shown that nilotinib treatment can induce a high rate of sustained and DMR and can lead to successful attempt at a second TFR in patients who previously failed a first discontinuation of imatinib (19).

When considering a second attempt at TFR, current recommendations are to restart with the same drug and consider possible discontinuation after obtaining a sustained, long-lasting and deep response for almost 2 years. For more detailed requirements, the reader is referred to the European LeukemiaNet 2020 recommendations (7).

### Second-Generation Tyrosine Kinase Inhibitors and Treatment-Free Remission

The most recent trials that led to the approval of second-generation TKIs in first line therapy (DASISION and ENESTnd) showed that nilotinib and dasatinib are associated with a much greater depth of response compared to imatinib (MR4.5 at 5 years were 54% vs 31% and 42% vs 33%, respectively) (20, 21). A very recent analysis of ENESTfreedom has also suggested that discontinuation of nilotinib may be feasible in elderly patients with CML in chronic phase, even if the Kaplan-Meier estimate rate of treatment-free survival at 5 years was somewhat lower in those ≥65 years (51.0% for <65 years vs. 37.5% for ≥65 years) (22). Moreover, in a 5-year update of TFR following second-line nilotinib (ENESTop trial), the long-term durability and safety of nilotinib re-initiation was demonstrated, and the majority of patients re-initiating nilotinib retreatment rapidly regained MR4.5 (23).

SUSTRENIM (NCT02602314, currently recruiting), a phase IV, randomized (1:1) interventional study with two arms, carried out in *de novo* CML in chronic phase, is the first trial to directly compare a second-generation TKI (nilotinib) to imatinib. The SUSTRENIM trial is enrolling newly diagnosed patients, but compared to ENESTnd allows a possible early switch from imatinib to nilotinib at 3 and 6 months in case of non-optimal response with the final aim to discontinue the treatment. It should be noted that in the imatinib group, in case of lack of optimal response (according to 2013 European Leukemia Network criteria), a crossover to nilotinib is foreseen as this reflects current clinical practice.

The STOP 2G-TKI study found that patients treated with a first-line second-generation TKI or who switched to second-generation TKI for intolerance have a better response than those who were resistant to a first-line TKI (24).

In addition to the results from randomized clinical trials, the observational study by Khoury et al. in patients who discontinued therapy with a TKI (imatitinib, dasatinib, or bosutinib) upon the patient’s request or for intolerance to therapy reported that 41% of patients lose MR3, while 59% maintain TFR without therapy (25). Moreover, an observational study in Italy by Fava et al. showed that a significant difference in TFR was seen in favor of a second-generation agent over imatinib after interruption of a TKI (26).

## The Importance of Monitoring

Quantitative molecular monitoring of the BCR/ABL fusion transcript is a crucial component of all stages of patient management in CML. During treatment with a TKI, it has been observed that one to two qPCR controls per year reduce the risk of progression or death by almost 60% and that this risk is reduced by an additional 50% with three to four annual controls (27). In addition, patient monitoring is essential to prevent QoL from reaching a critical threshold below which the risk of poor adherence increases (28). On the other hand, optimal therapy requires optimal monitoring, which has both clinical and economic benefits (27, 29).

Monitoring is also essential to verify maintenance of DMR, which is a necessary requirement to initiate TFR, in agreement with the eligibility criteria of all clinical studies on TFR. Finally, close monitoring during TFR (every month for the first 6 months and then every 6–8 weeks for the first year) can reassure physicians and patients, allowing prompt intervention in case of loss of MMR and prevent overt recurrence of disease. In this regard, the rapidity of laboratory referrals is important, and the presence of a network of laboratories is very useful in providing timely diagnoses. Recently, more sensitive techniques for transcript evaluation (digital PCR) have been introduced that can positively impact patient selection and help predict the success of TFR (3, 30).

## Management of Discontinuation-Related Adverse Events

The effects of TKI discontinuation are not homogeneous. Although generally transient, the possibility of side effects following discontinuation of a TKI must be explained to the patient. With TFR, a withdrawal syndrome has been observed that causes adverse events such as muscle pain and arthralgia (31). Among patients in the EURO-SKI study, 29.7% and 1.2% experienced grades 1–2 and grade 3 muscle pain, respectively. In mild cases, the use of paracetamol or NSAIDs was sufficient to resolve the pain, while in some patients it was necessary to administer corticosteroids for a prolonged period (31).

## Markers for Treatment-Free Remission

CD26, a marker of circulating stem cells from CML, is an important marker in patients who have to stop treatment (32). Most patients who have achieved TFR maintain detectable levels of CD26+ cells, which could indicate the presence of circulating leukemic stem cells; an inverse linear correlation has been demonstrated between the number of circulating CD26+ cells and duration of TFR (32).

Several studies have shown that natural killer (NK) lymphocytes can be used as marker to predict the success of TFR. In particular, the IMMUNOSTIM study reported that a low number of NK cells were present at the time of discontinuation in patients who relapsed (33).

The EURO-SKI study highlighted the role of another relevant marker for TFR, namely CD56. In fact, in patients without recurrence, increased levels of NK with cytotoxic phenotype (CD57+, CD56dim) and interferon/tumor necrosis factor (IFN/TNF) were seen at the time of discontinuation compared with those who relapsed (16). Thus, several markers are available that can be used to monitor TFR.

## Non-Conventional Treatment Schedules Under Study

Several studies have examined the use of non-conventional treatment schedules to further improve the possibility of achieving TFR. In a single-arm study, Nicolini et al. combined nilotinib with pegylated interferon (NiloPeg), which was relatively well tolerated and associated with a high rate of MR at one year (17% of patients with MR4.5) (34). These results were confirmed in a recent randomized study presented at the American Society of Hematology (ASH) annual meeting in 2019, which reported an MR4.5 response at 36 months in 49.5% of patients treated with NiloPeg and in 37.2% of those treated with nilotinib; there were no marked difference in the safety profiles of the two study arms (35). The DANTE study (NCT03874858, currently recruiting) is examining the use of nilotinib at half the standard dose (from 600 to 300 mg) prior to TFR (48-week consolidation phase) in patients with sustained DMR to evaluate if the treatment schedule can be optimized.

## Key Points for Clinical Issues Related to Treatment-Free Remission in Chronic Myeloid Leukemia

In light of the above, DMR can now be considered as the primary goal of treatment, independently of whether the aim is to control the disease or to achieve TFR. Second-generation TKIs ensure that DMR is achieved rapidly, and are more successful in attaining TFR compared to first-generation TKIs. When attempting TFR, monitoring every 3 months is needed before discontinuation of the TKI; after interruption, patients should be monitored every month for the first 6 months and every 6-8 weeks starting at month 7 after discontinuation. The loss of MMR does not define recurrence of disease, but, through monitoring, it does allow the clinician to intervene promptly and prevent it. Indeed, even if half of patients will lose MMR sooner or later, it is possible to successfully restart treatment, achieve MMR again and even make a second attempt at TFR.

## Discussion

### Factors to Consider When Proposing Treatment-Free Remission

The possibility of TFR should be mentioned to the patient at diagnosis or at least during initial visits. Rendering patients aware of the possibility of TFR at diagnosis can be useful, especially in young patients with a higher socio-cultural profile, as it may help improve adherence to therapy. TFR should be perceived as an attempt to verify whether it is possible to maintain a MMR in the absence of treatment. Rigorous follow-up must be presented as an opportunity that provides greater reassurance, with the aim of promptly identifying loss of response and allowing for early re-initiation of therapy. The possibility of loss of MMR must be clearly indicated to the patient as an event that occurs in a significant proportion of patients. However, it should also be stressed that loss of MMR does not mean that the disease has recurred as at the first diagnosis. In fact, the response can be regained by restarting therapy, even if this is achieved at different times with great variability depending on the patient. Patients should also be counseled about the possibility of adverse events following discontinuation, which may be long-lasting or transient. Thus, TFR should be viewed as a shared pathway based on an alliance between the patient, physician and treatment center.

### Aspects to Avoid When Proposing Treatment-Free Remission

Above all, lack of empathy should be avoided. The physician should speak the patient’s language, and not cite clinical studies, study groups, percentages, or data if the patient’s educational level does not allow him/her to understand the information. At the same time, simplified language should not be used with more knowledgeable patients. Attention should be paid to both verbal and non-verbal language. Phrases and words that can create anxiety if loss of MMR occurs during TFR should be avoided. In addition to being clinically incorrect, such language is unnecessarily intimidating and conveys a lack of belief and inadequate experience of the clinician in TFR. The patient should be strongly encouraged to attempt TFR if the clinical conditions permit it. In short, TFR should be viewed as a possibility for healing.

### Treatment-Free Remission in Special Settings

Special categories of candidates for TFR include those with side effects and concomitant pathologies as well as all young women who become pregnant. Concerning this latter category, it should be noted that TKIs can be teratogenic, and thus their use is contraindicated in pregnancy, and especially during organ formation (36). It is possible for women with CML to plan a pregnancy, and access TFR before conception, or stop TKIs as soon as pregnancy is discovered, before organogenesis, although pregnancy must be carefully evaluated in case of advanced disease, after multiple treatment lines, since response to discontinuation of therapy is generally not favorable (37). TKIs should not be used during pregnancy unless the clinical condition requires treatment. Imatinib and nilotinib have limited placental transfer, and as such their use can be considered after placental formation and crucial fetal organ development (15-16 weeks) (38). Lastly, interferon can be used at any time during pregnancy (39).

### Concluding Remarks

TKIs have undoubtedly dramatically improved management of CML and form the mainstay of treatment. However, some patients may develop resistance or intolerance to TKIs and a number of agents with different treatment targets are currently under investigation [reviewed in (40, 41)]. Herein, the attempt was made to define the challenges that clinicians face when considering TFR from both clinical and psychological points of view. Based on the outputs from the expert participants, a list of inadequacies and possible solutions are proposed herein that may help those with less experience in CML and TFR in daily practice. The overall objective is to render both patients and clinicians more confident with the prospect of discontinuing therapy. Patients should be made aware of the possibility to attempt TFR as a part of routine therapy with the realization that TFR may not be achieved in all patients, and may not necessarily be long lasting even if achieved. It is reiterated that the patient is knowledgeable about the need for increased monitoring, and that loss of MMR does not equate with overt relapse. Therapy can be restarted and MMR achieved. Women patients should be aware that pregnancy is possible in many cases with no adverse effects on the infant. Lastly, the results of our survey indicate that better education of patients and clinicians is warranted.

## CML-TFR-ITA Working Group Contributors

Silvana Annunziata, Francesca Celesti, Giuseppe Cimino, Giuseppina De Falco, Marianna De Muro, Maria Rosaria Esposito, Luca Franceschini, Domenica Gangemi, Maria Iovine, Gaetano Labarba, Sabrina Leonetti Crescenzi, Valentina Maglione, Fausto Palmieri, Giuditta Pollio, Raffaele Porrini, Anna Maria Rauco, Antonello Sica, Federica Sorà, Francesca Spirito, Roberto Vallone.

## Author Contributions

All authors contributed to the article and approved the submitted version.

## Conflict of Interest

MB received honoraria by Novartis, Pfizer, Incyte, BMS/Celgene. EA has served as Advisor or Consultant for BMS, Incyte, Novartis, Pfizer and Takeda.

The remaining authors declare that the research was conducted in the absence of any commercial or financial relationships that could be construed as a potential conflict of interest.
